# Dr. Clinton Colgrove

**Published:** 1906-12

**Authors:** 


### OBITUARY.

Dr. Clinton Colgrove died at Holland, N. Y., October 19, 1906, in the eightieth year of his age. He was born at Sardinia, Erie County, N. Y., June 9, 1827 and was the son of Dr. Bela H. Colegrove, a surgeon of rare ability who had an extensive practice extending over several counties of western New York and into Pennsylvania. Dr. Clinton Colegrove spent several years at Madison (now known as Colgate) University and then came to Buffalo to take up his medical studies. He graduated at the medical department, University of Buffalo, in 1850 and began to practise at Sardinia, N. Y. Later he removed to Fredonia, but upon the death of his wife some years ago he moved to Arkansas, thence to Westfield, Mass., and spent his closing years at Holland. His mother was a sister of William Ives of Buffalo and of the late Dr. James Ives of Strykersville.

Dr. Colegrove was a scholarly man, of great refinement and purity of character. He was eminent rather as a writer than as a practitioner and contributed many articles in prose and verse to the secular and religious press. He was a man of poetic instincts and his writings excel along these lines.

